# Effectiveness of a Culturally-Tailored Smoking Cessation Intervention for Arab-American Men

**DOI:** 10.3390/ijerph14040411

**Published:** 2017-04-13

**Authors:** Linda G. Haddad, Ahmad M. Al-Bashaireh, Anastasiya V. Ferrell, Roula Ghadban

**Affiliations:** 1 College of Nursing, University of Florida, Gainesville, FL 32610, USA; aalbashaireh@ufl.edu (A.M.A.-B.); amitryukhina@ufl.edu (A.V.F.); 2 School of Nursing, Virginia Commonwealth University, Richmond, VA 23824, USA; ghadbanr@vcu.edu

**Keywords:** smoking cessation, NRT, Arab American, cultural, linguistic, tailoring

## Abstract

To date, no smoking cessation programs are available for Arab American (ARA) men, who are a vulnerable population with high rates of smoking. Thus, the primary aim of this one group pre-test/post-test study was to assess the effectiveness of *Sehatack*—a culturally and linguistically tailored smoking cessation program for ARA men. The study sample was 79 ARA men with a mean age of 43 years who smoked between 5 and 40 cigarettes (mean = 19.75, SD = 9.1) per day (98.7%). All of the participants reported more interest in smoking cessation post-intervention and many of the participants in the baseline (38.5%) and post-intervention phases (47.7%) wanted to quit smoking ”very much”. For daily smokers who completed the smoking cessation program, the median number of cigarettes smoked daily was significantly lower than those in the post-intervention phase (Z = −6.915, *p* < 0.001). Results of this preliminary study indicate that: (a) *Sehatack* may be a promising way for ARA men to quit smoking, and (b) culturally relevant smoking cessation counselors can be trained to recruit and retain ARA smokers in an intensive group smoking cessation program. Strengths of this study were community engagement and rapport between three faith organizations and the University of Florida College of Nursing. However, a larger trial is needed to address study limitations and to confirm benefits in this population.

## 1. Introduction

Despite the fact that this population totals almost six million and is one of the fastest-growing immigrant groups in the United States [1,2,3,4,5], there have been few studies of smoking behavior among Arab American (ARA) men, who often have high rates of smoking. Indeed, the prevalence of cigarette smoking among men in the Middle East is very high (63% men, 10% women) [6,7] and once in the United States, the prevalence is approximately 20%–30% higher among ARA men than among the general U.S. adult male population. ARA men also have a lower quitting ratio compared to U.S. adult males [8,9,10,11,12,13], perhaps because many ARAs come from countries where tobacco use among men is culturally appropriate. These high smoking rates present serious health risks to ARA men, who are at increased risk for chronic diseases, including a variety of cancers, and higher mortality rates [14,15]. In addition, their families, which tend to be large and include non-immediate family members, are also at risk for chronic health issues because of exposure to secondhand smoke [15]. Despite these serious health risks, to date, no smoking cessation program is available for Arab American men.

Thus, to fill this gap in research, we have created a novel, culturally tailored and linguistically appropriate smoking cessation program for ARA men in order to examine changes in smoking behavior, and ultimately, to reduce rates of smoking in this at-risk population. Arabic-speaking men have been influenced by cultural tradition, intense tobacco marketing, and weak tobacco control laws in their native lands [16,17,18]. Our culturally-tailored intervention is titled, *Sehatack*, which is an Arabic word that means ‘your health’. *Sehatack* has been culturally tailored to reflect Arab values and cultural beliefs from the male perspective. Like many other successful smoking cessation programs in the United States, *Sehatack* includes motivational interviewing (MI) strategies built upon the Transtheoretical Model (TTM) [19], along with telephone counseling in Arabic, pretested workbooks in Arabic, and nicotine replacement therapy (NRT). We acknowledge that interventions such as MI, NRT, behavioral feedback and telephone counseling are not novel and are recommended in current Clinical Guidelines [20,21,22,23]. However, we adapted the intervention culturally and linguistically for ARA men to create a novel, integrated intervention that is culturally tailored to this minority group. In our pilot work, we have found that ARA men are interested and willing to embark upon smoking cessation when they are offered a culturally tailored, linguistically appropriate program. We have also found that religious leaders in the Arab community welcome our research program in their mosques and churches, where we recruited our participants.

It should be noted that Arab American men interested in reducing and/or eliminating smoking often experience a greater disparity in joining smoking cessation programs. This occurs for several reasons: first, many are underemployed and must work long hours to support their large families [24]. Thus, they may not have time to attend groups or classes on smoking cessation. In addition, ARA men may be socially marginalized in health care or support group situations due to stereotypes stemming from terrorist attacks in the US and geopolitical unrest in the Middle East [25,26]. Finally, newly immigrated ARAs find it difficult to participate in typical cessation programs because they are not sufficiently fluent in English [27]. This language barrier persists long after immigration because most ARAs depend upon Arabic for religious practice in Mosques as a well as Orthodox churches (e.g., Maronite and Coptic) [15]. Therefore, to address the disparity of tobacco-related health issues for ARA men, effective smoking cessation programs must reflect the culture, values, beliefs and language of this group.

To enhance the effectiveness of tobacco interventions among minorities, researchers have called for cultural adaptation and tailored tobacco interventions [15]. ‘Targeted’ interventions focus on expanding the generalizability of an intervention (developed in other populations) to minority groups, and while these interventions may have embedded dominant cultural values, they do not take the values and experiences of a minority group into consideration [15,28]. In contrast, ‘tailored’ interventions focus on ensuring that the intervention meets the unique needs of the minority group by including ethnic/cultural experiences, norms, and values [29,30,31,32,33]. To our knowledge, existing reviews of cessation studies have not yet addressed the cultural values and experiences of the ARA minority group. Therefore, the purpose of this study was to examine the preliminary effectiveness of a culturally and linguistically tailored smoking cessation program for ARA male smokers.

Aim: In this study, we aimed to assess the effectiveness of “*Sehatack*”—a culturally and linguistically tailored smoking cessation program for ARA men.

Primary hypothesis: Compared to pre-test baseline measures, participants’ post-intervention measures will demonstrate the following changes in the primary outcome variables: (1) successful smoking abstinence; (2) increased number of quit attempts; (3) reduced number of cigarettes smoked per day and per week.

Secondary hypothesis: Compared to pre-test baseline measures, participants’ post-intervention measures will demonstrate: a reduction in perceived withdrawal symptoms, and an increase in quitting self-efficacy in both short-term and long-term follow-ups.

## 2. Materials and Methods

The study was approved by the University of Florida institutional review board prior to commencement and was conducted between 2015 and 2016. It is important to note that this study was not registered in Clinical trial.gov.

### 2.1. Population and Study Sample

**Figure 1:**
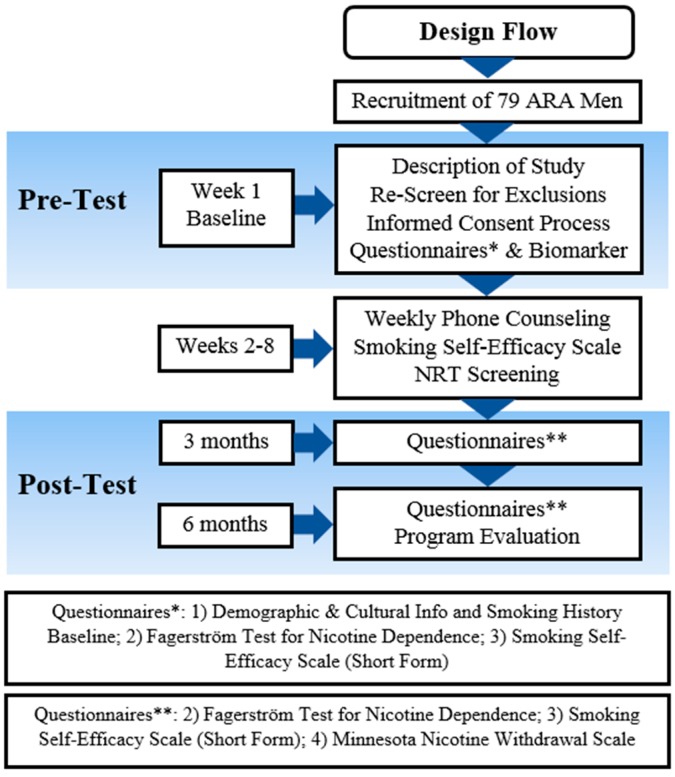
Study Timeline. (ARA: Arab American).

We had a one-group pre/post-test design as a pilot study for *Sehatack*, a culturally and linguistically tailored smoking cessation program for ARA men (see Figure 1). We recruited a non-random sample of 80 self-identified ARA male smokers. Inclusion criteria: Potential participants were required to: (1) be adult men (18–65 years of age); (2) self-identify as ARA; (3) have smoked or puffed on a cigarette during the previous week; (4) have an exhaled carbon monoxide (ExCO) reading of 7 ppm or higher as an indicator of current smoking; (5) be able to read, write, and speak Arabic; (6) be willing to participate in the smoking cessation study; (7) have telephone access; (8) be present in the study geographical area for 6 months; and (9) have never been enrolled or not be currently enrolled in another smoking cessation treatment program. The fifth criteria was not restrictive given that the recruitment settings offered their services in the Arabic language, and 80% of our previous studies’ participants were able to read, write, and speak Arabic. Exclusion criteria: Potential participants should not: (1) have had a plan to move out of Florida within 6 months following study enrollment; (2) have had a myocardial infarction in the two weeks prior to study commencement; or (3) have been hospitalized for a heart-related condition in the two weeks prior to study commencement.

### 2.2. Data Collection Procedure and Sites

The study team has a history of successfully recruiting and retaining Arab Americans into smoking cessation programs. In this study, both active and passive recruitment approaches were used along with a community outreach method. Active recruitment approaches consisted of outreach at locations where members of the Arab American population socialize or congregate; these locations included festivals, mosques, churches, community groups, and Middle Eastern grocery stores. Passive community outreach consisted of posting flyers at venues popular with the community, personally referring an eligible participant to the study, and spreading awareness of the study through word of mouth advertising.

Around 180 potential participants responded to advertisements posted on social media as well as fliers distributed in Middle Eastern grocery stores, restaurants, lounges, and faith-based organizations in four cities in Florida: Jacksonville, Tampa, St. Petersburg, and Orlando. The ads invited Arab Americans to participate in a smoking cessation study and listed a phone number they could call if they were interested in participation. Other potential participants were approached by a member of the study staff in places where social interaction was possible, such as an Islamic cultural center. Data collection occurred at mosques, churches and cultural centers that were frequented by ARA men as well as at colleges/universities, Middle Eastern grocery stores, restaurants, and lounges. Potential participants were identified through a brief screening process upon response to the ads and flyers. During the screening process (either by phone or face-to-face meeting), potential participants were directed to the study PI and/or research assistant. Interested participants were asked to attend a screening session, during which they were told about the study and were given an opportunity to provide consent if they were interested. Participants were then asked a series of screening questions to determine their eligibility for the study, as well as demographic questions. After screening and consent, participants were asked to complete a contact form, and then complete a series of questionnaires. Out of the 115 men who were screened 79 men met the eligibility criteria. Whereas 36 men were excluded as 10 men were originally from the Middle East but not Arab countries so did not speak or read Arabic, 15 men have plans to move from Florida, and 11 men have ExCO reading below 7 ppm.

### 2.3. Intervention Components

(1). The Sehatack Self-Help Workbook Protocol has been designed on the basis of MI within the TTM framework. The *Sehatack* self-help workbook consisted of four parts (or stages). Stage 1—contemplation (Week 2): The information in Stage 1 of the *Sehatack* guide was geared toward smokers who were considering smoking cessation and explored the cost/benefit analysis of quitting smoking and the obstacles that prevent participants from quitting. Stage 2—preparation (Weeks 3–4): During Stage 2, participants developed a plan to stop smoking. The material in the *Sehatack* Smoking Cessation Guide focused on physical and mental preparation for smoking cessation, why it was important for participants to quit, how smoking impacted their health, and how family members and friends could support a quit attempt. Stage 3—action (Weeks 5–6): This stage was designed for participants who quit smoking during Stage 2. The focus during this stage was on helping participants manage withdrawal symptoms and deal with smoking triggers. Stage 4—maintenance (Weeks 6–8): The final stage of the workbook was designed to help participants maintain their smoke-free status. More details about how the intervention was adapted is previously published by Haddad ad Corcoran [34]. Participants received information on how to deal with cravings and how to continue living smoke-free, including a discussion of relapse prevention strategies.

For each stage, the workbook contained homework, such as developing personalized strategies for dealing with high-risk situations and avoiding relapse as well as what to do if a relapse were to occur. The workbook also taught problem solving and relapse prevention skills (e.g., recognizing and coping with cues that could precipitate relapse to tobacco use); provided skills training (e.g., lifestyle changes, and relaxation techniques); and gave basic information about the harmful effects of tobacco, the benefits of quitting, and nicotine withdrawal symptoms. All problem solving and skills training were designed on the basis of Arabic cultural values and assumptions, such as deep religious orientation, reliance on the extended family, defined gender roles and taboos, use of the Arabic language, and adherence to traditional beliefs and practices. For greater impact, social and family concerns were emphasized in the intervention [15].

(2). Telephone Counseling (TC): Reviews of TC and intensive smoking cessation behavioral interventions concluded that they were equally effective in enhancing quit rates [35,36]. In this study, two interventionists/counselor conducted four individual telephone-counseling sessions (30 min each) at the middle of each stage over an 8-week period (see Figure 1). TC session content was primarily drawn from MI techniques to increase the participant’s motivation to change. Session topics included coping with withdrawal, maintaining a commitment to continued abstinence, and relapse prevention. Each participant was assigned to the same counselor (who made all calls to that participant) in order to optimize rapport and familiarity with the participant’s unique concerns and smoking history. Two male Arab American were hired for the purpose of prepare them to conduct the telephone counseling. The two counselors are first generation American so Arabic is their first language. One of them is originally Syrian and the other is originally from West bank.

The study PI and MI consultant, who are bilingual Arabic speakers, trained two counselors/interventionists in telephone counseling as well as Motivational Interviewing. The PI was responsible for monitoring treatment and controlling intervention fidelity and consistency. Throughout the study, interventionists were supervised by objective experts and the study PI and director, whom they met with every 2 weeks to discuss issues and to prevent drift. Adherence checklists were also employed to monitor treatment fidelity and to periodically review the interventionists’ performance for consistency and accuracy.

(3). Nicotine Patches: Over-the-counter nicotine patches were given at baseline (week 1). The research assistants reviewed the procedures for obtaining and using the NRT (patches) with all study participants, if requested, free of charge for up to 8 weeks. During the first visit, participants who chose to use NRT were provided with a two-week supply of NRT, and could obtain up to 6 more weeks of NRT free of charge after each two-week follow-up call. The patches were shipped to the participants after confirming participant’s need for specific nicotine concentration (i.e., 7 mg, 14 mg, or 21 mg). Participants’ need for NRT was decided based on their responses to the “NRT Screening Tool and Dosing Guide” form (see Supplementary Materials).

### 2.4. Measures

The measurement instruments that were used in this study have been tested and validated in ARA communities [7,9,34]. The main study outcomes were measured at baseline, at weeks 2, 8, and 12, and at 6-month time points as follows: (1) Demographics, culture and smoking history form were used to obtain background and cultural information, including a 7-item Arab Acculturation Scale, a measure of smoking history, smokeless tobacco use, smoking habits, past attempts to quit smoking, and the desire to quit; (2) Fagerström Test for Nicotine Dependence (FTND), a 6-item scale that was used to measure the level of nicotine dependency or addiction. The total score of the 6 items ranged between 6 and 16 and was categorized as follows: 6–10, 11–12, and 13–16 as high, medium, and low nicotine dependence, respectively. The Fagerström Test has high test-retest reliability (*r*’s ranging 0.70–0.88) [37,38,39,40,41]; (3) The Perceived Self-efficacy/Temptation Scale is a reliable and well-validated (alphas: 0.8–0.9) 9-item self-efficacy scale that was applied to measure the participant’s confidence in his ability to abstain from cigarette smoking in a variety of different situations [42,43,44]. The total score of the 9 items ranged between 9 and 45 and the higher score meant higher self-efficacy to quit smoking; (4) Minnesota Nicotine Withdrawal Scale (MNWS) is a 15-item scale that measured smoking withdrawal symptoms, including anger, anxiety, cravings, depression, difficulty concentrating, hunger, impatience, insomnia, and restlessness [45,46]. The total score of the 15 items ranged between 15 and 75 and the higher score mean more severe withdrawal symptoms. The MNWS has high internal validity (alphas: 0.80–0.90) and high test-retest reliability (*r*: 0.64–0.71) [41,47,48]; (5) Program Evaluation Questionnaire is an 8-item questionnaire that was used to assess the participants’ perceived satisfaction with *Sehatack* program components [34]; (6) Smoking Cessation and Reduction was assessed by asking participants whether they have smoked or had a puff on a cigarette in the past 7 days and the number of cigarettes smoked in the past 7 days. Participants’ self-reported number of cigarettes smoked at baseline, and at 2-week, 8-week, 12-week, and 6-month time points were used as a measure of smoking reduction. Measurement of cigarettes per day has high test-retest reliability (*r* = 0.91) [41]; (7) Biomarker of Smoking Status: Carbon monoxide biochemical marker was used to check participants’ carbon monoxide exposure, which can occur during cigarette smoking. Carbon monoxide (CO) concentration in parts per million (ppm) from expired breath was measured using a Micro CO meter (Micro Direct Inc., Lewiston, ME, USA). An ExCO reading of 6 ppm or higher was used as an indicator of current smoking [49,50]. ExCO was measured as cross validation of participants’ smoking status at baseline. The test-retest reliability of the carbon monoxide measurement was previously reported as slightly low (*r* = 0.67) [41].

### 2.5. Statistical Analysis

IBM SPSS Statistics version 24 was used to analyze the data [51]. Frequency and percentages were provided for categorical data. Continuous data were described in terms of the mean and standard deviation. Essential characteristics of completed cases were compared side-by-side with participants lost to follow-up. Mean and standard deviation were reported for normally distributed baseline and post-intervention values, while median and quartile were reported for non-normally distributed data. If the data were normally distributed, a paired *t*-test was used to study the changes in the mean in two point measurements. Repeated Measure ANOVA, within groups, was used in case of three or more points of measurement. Bonoferroni adjustment for level of significance was applied in multiple pairwise comparisons. Wilcoxon Sign Ranked Test was used to compare the differences in median within the group between any two given points of measurement (e.g., baseline and post intervention). A Friedman test was also used to test the changes in median within the group if there were more than two points of measurements.

Pre-and post-intervention, the number of cigarettes smoked per day, and the number of cigarettes smoked per week, were analyzed using intent to treat analysis with last observation carried forward. Fourteen subjects were missing in the post intervention data for these two measures. The baseline data for these measures for these 14 participants were used as the value of the post intervention measure. Also, when the participant stopped smoking was analyzed both pre-and post-intervention using intent to treat analysis with the last observation carried forward. All hypotheses were tested as two-sided, with a level of significance of *p* value ≤ 0.05.

## 3. Results

### 3.1. Baseline Data

**Figure 2:**
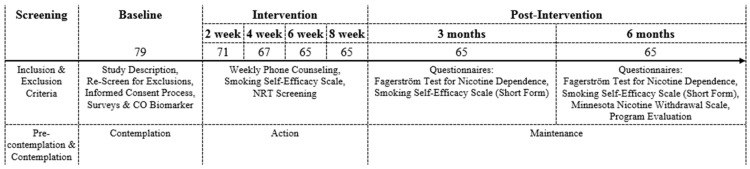
The number of participants across the phases of the study.

This study enrolled 79 Arab-American men living in the Central North Florida. Figure 2 provides more data about the number of participants across the different phases of the study. Table 1 describes demographic characteristics of the study sample. Participants’ age ranged between 18 and 64 years (mean = 43.2, SD = 10.7). The majority of the 79 participants were married (81%) and employed (78.4%). Many of the recruited 79 men had at least high school education (29%) and/or some level of college degree (55.7%). Out of the 79 of participants, 76 (96.2%) shared their parents’ birth country. Out of the 14 represented countries, most of the participants were from Egypt (24%), Iraq (17.7%), Palestine (16.4%), and Syria (12.6%).

The majority of the participants were daily cigarette smokers (98.7%) with exhaled CO ranging between 7 and 41 ppm. Table 2 shows participants’ baseline smoking history. The number of cigarettes smoked ranged from 5 to 40 cigarettes a day (mean = 19.75, SD = 9.1) and 20 to 450 cigarettes a week (mean = 155.9, SD = 83.9). Although most of the recruited men had heard of e-cigarettes before the study (72.2%), half of them (49.4%) did not consider e-cigarettes before the study (49.4%) and a third had already used e-cigarettes in addition to conventional cigarettes (30.3%) before the study. Out of the 79 participants, 62 (78.5%) had previously attempted to quit smoking and 54 (68.4%) had previously attempted to quit over the last two years. The majority of these attempts consisted of quitting cold turkey (38%). More than half of the participants admitted that these attempts were difficult (26.6%) or very difficult (34.1%). Many of the participants had no trouble refraining from smoking in public places (64.6%) and were confident in their ability to quit smoking within a year (83.5% rated own self-efficacy more than 5 out of 10). Health concern was the most influential in participants’ decision to quit or reduce smoking (73.4%). While a majority of the participants had family rules against smoking inside or near their homes (86.1%), many of the recruited men were the primary smokers in the family (86.1%) and were exposed to tobacco smoke at home (75.9%).

Out of the potential participants who were in the Pre-Contemplation and Contemplation stages, the 79 recruited men were in the Contemplation stage (refer to Figure 2). The 14 men who were lost to follow-up after the first week of study skipped the Contemplation, Preparation, and Action stages to move to the Relapse stage. Eight cases were lost to follow-up at week 2, four cases at week 4, and two cases at week 6. Remaining 65 participants moved from the Action to Maintenance stages. Table 3 outlines the characteristics of people lost to follow up and compares them to the completed cases. Table 4 provides the total score of Minnesota Nicotine Withdrawal Scale (MNWS) for responses from lost and completed cases in weeks 2 and 4.

### 3.2. Primary Outcomes: Smoking Cessation and Reduction

#### 3.2.1. Smoking Abstinence and Number of Quit Attempts

Many of the participants either continued occasional smoking (40%) or stopped daily smoking two to six days prior to answering the questionnaire in the post-intervention phase (43.1%). Table 5 shows results of the Smoking Cessation and Reduction scale. All of the participants reported more interest in quitting smoking post-intervention. Almost half of the participants reported they wanted to quit smoking “very much” (47.7%). Almost all participants were confident they would not be smoking one year after the intervention (98.5%). The majority of respondents reported that they either will quit (33.8%) or maintain quitting (44.6%) in the next month. Many participants in the baseline (38.5%) and post-intervention phases (47.7%) wanted to quit smoking “very much”.

Using intent to treat with last observation carried forward to account for missing data, there was a statistically significant difference between the ranked when the participant stop smoking daily smoking pre and post intervention (Wilcoxon Signed Rank Z = −8.85, *p* < 0.0001) (see Table 6). The results of both tests remained statistically significant even when the analysis was limited to participants who completed the study.

#### 3.2.2. Number of Smoked Cigarettes per Day and per Week

Using intent to treat with last observation carried forward to account for missing data, there was a statistically significant difference between the number of cigarettes smoked per day both pre- and post-intervention (Wilcoxon Signed Rank Z = −5.8391, *p* < 0.0001). The number of cigarettes smoked in the last week post intervention was statistically significantly different from the number of cigarettes smoked in the pre-intervention measure (Wilcoxon Signed Rank Z = −8.1274, *p* < 0.0001) (see Table 7). The results of both tests remained statistically significant even when the analysis was limited to participants who completed the study.

### 3.3. Secondary Outcome: Withdrawal Symptoms, and Quitting Self-Effiacy

Responses to the Minnesota Nicotine Withdrawal Scale (MNWS) from the completed cases show little change in participants’ mean withdrawal symptoms (scores range 15.1–16.5), mean heart rate (range 82.1–82.3 bpm), and mean body weight (80.7–81.1 kg) between weeks 2 and 27. No statistical significance was noted across the intervention (weeks 2, 4, 6, and 8) and post-intervention (weeks 14 and 27) time points.

Participants’ adherence to the NRT use was self-reported as 97.5% (77 out of 79 people used the patches). Participants reported the following adverse events after the nicotine patch use: mild irritation, mild pain (1–3 out of 10), and headaches.

Compared to the baseline participants’ self-efficacy to quit smoking remained constant in the short- and long-term (9, 9, 9, respectively; Friedman *p*-value = 0.155).

### 3.4. Overall Impressions of Intervention

The overall satisfaction about the *Sehetack* program was calculated based on four likert scale items: materials were helpful, materials were personally relevant, the program met your expectations, and you will use this program in the future if you have a chance. All participants answered the four items with a response of ‘agree’ or ‘strongly agree’. The mean for the rate of overall satisfaction was 93%.

## 4. Discussion

To our knowledge, this is the first study evaluating the effectiveness of a culturally and linguistically tailored smoking cessation program for ARA smokers. Consistent with other studies, the NRT helped reduce withdrawal and addiction symptoms related to tobacco [34].

The combination of Nicotine Replacement Therapy (NRT) with other smoking cessation strategies is superior to using NRT alone for short-term smoking cessation [52,53,54]. Our findings suggest that the combination of NRT with Motivational Interviewing (MI) may be a promising way to treat tobacco dependence in smokers, and it appears to be safe and well-tolerated. However, it should be noted that given the high abstinence rates for NRT alone on both short-term and long-term outcomes, the effectiveness of the MI and the nicotine patch employed in this study appears to be maximal, suggesting that the NRT is the major contributor to the participants’ smoking cessation and the MI has only a supplementary effect. Future studies should include randomized control of the NRT and MI in the ARA population.

For most smokers, symptoms of tobacco withdrawal were relatively brief, with most symptoms decreasing below baseline within one to three weeks of quitting smoking. There was a decrease in the nicotine dependency scores among participants from baseline to post-intervention phase 1 (after three months) and post intervention phase 2 (after six months). Significantly, after eight weeks of follow-up telephone counseling and transdermal nicotine therapy, this study revealed that even smokers who did not quit smoking post-intervention decreased their daily intake of cigarettes from 20 to 4. This finding indicates that behavioral interventions, such as self-help materials, therapist-delivered interventions, and intensive individual or group counseling, may help relieve tobacco dependence and aid smoking cessation [55,56].

Collectively, preliminary results from this study suggest *Sehatack* may help ARA men reduce and/or quit smoking. Indeed, the end-of-treatment smoking cessation rate was 43.1% after six months, which is substantially better than the quit rates among African American (range 11.2%–27.5%), and Alaska Native smokers (30%) [15,28] who have completed smoking cessation programs. A possible contributor to this difference may be a genetic disposition in these populations, a variance in study design and conduction, or specific social factors attributed to the cultures of these populations [57,58,59,60]. Participants reported high degrees of satisfaction with the program as a whole. Thus, culturally tailored and linguistically appropriate intervention approaches may be more effective than standard interventions, because the former considers the sociocultural characteristics of the target minority population.

Results of the preliminary study demonstrated that it is possible to successfully recruit and retain Arab American men into a smoking cessation program as well as train counselors who can work effectively with this vulnerable population. The strengths of this study were the community engagement and rapport between three faith organizations and the University of Florida College of Nursing. For example, a Lebanese pastor at a church in Tampa supported data collection at a Sunday lunch and offered the church as setting for regular collaboration with the community.

### 4.1. Future Research

Other studies are needed that examine the feasibility and effectiveness of socioculturally relevant smoking cessation programs in ARA communities. Specifically, future research should focus on incorporating participant feedback from the current study as well as evidence from reviewed literature to determine strategies that will enhance quit rates among ARA smokers.

### 4.2. Study Limitations

While the study findings are encouraging, the study did not provide long-term abstinence rates for participants. (The gold standard for smoking cessation outcomes is 1-year of abstinence). In addition, the sample size includes only 65 men; thus, future studies should include a larger sample size and follow participants for at least one year. Also, a self-reported reduction and cessation instrument was used without any biological validation. This may have resulted in recall bias and inaccurate reporting. Finally, there was no randomized control group employed, so it is unknown if improvements were due to election, time, or other threats to internal validity.

## 5. Conclusions

To date, few culturally tailored and linguistically appropriate interventions exist for ARA men, who have very high rates of smoking [7,9,34]. Smoking cessation is the most important preventive health behavior ARA men can take to reduce their chances of a variety of smoking-related diseases. This study shows that, coupled with the NRT and MI, *Sehatack* appears to help ARA men reduce and/or quit smoking.

## Figures and Tables

**Table 1:** Demographic Characteristics (*n* = 79).

Variables	Mean (SD) or *n* (%)
Age (in years)	43.2 (10.7)
Marital status	
Married	64 (81)
Divorced	4 (5.1)
Never married	11 (13.9)
Schooling (in years)	14.7 (4.0)
Employment	
Working for pay	62 (78.4)
Unemployed and looking for work	12 (15.2)
Temporarily laid off or on leave	1 (1.3 )
Disabled/Unable to work	1 (1.3)
Retired	2 (2.5)
Student	1 (1.3)
Country of birth	
Algeria	1 (1.3)
Egypt	19 (24)
Iraq	14 (17.7)
Jordan	3 (3.8)
Lebanon	1 (1.3)
Libya	1 (1.3)
Morocco	5 (6.3)
Palestine	13 (16.4)
Somalia	1 (1.3)
Sudan	1 (1.3)
Syria	10 (12.6)
Tunisia	6 (7.6)
USA	3 (3.8)
Unknown	1 (1.3)
Age when moved to the US (in years)	29.3 (13.7)
Primary language(s) spoken at home	
English	2 (2.5)
Arabic	44 (55.7)
Other	33 (41.8)

**Table 2:** Baseline smoking history (N = 79).

Variables	Mean (SD) or *n* (%)
A: Smoking Status	
Current tobacco use	
Yes	79 (100)
Daily cigarette smoking	
Yes	78 (98.7)
Use of at least 100 cigarettes in a lifetime	
Yes	79 (100)
Number of daily smoked cigarettes	19.75 (9.1)
Number of cigarettes smoked in the past week	155.9 (83.9)
Frequently used products	
Manufactured cigarettes	78 (98.7)
Water-pipe	1 (1.3)
Products used in the past 3 months	
Manufactured cigarettes	78 (98.7)
Water-pipe	1 (1.3)
Awareness of e-cigarettes at the time of recruitment	
Yes	18 (22.7)
No	57 (72.2)
Uncertain	4 (5.1)
Prior consideration of e-cigarette use	
Yes, in addition to my other mode of smoking	24 (30.3)
Yes, but only by itself	4 (5.1)
No, I haven’t considered that before	39 (49.4)
No, I haven’t tried it and it didn’t appeal to me	9 (11.4)
I have not heard about it before	3 (3.8)
Primary mode of smoking	
Manufactured cigarettes	77 (97.4)
Self-rolled cigarettes	1 (1.3)
Water-pipe	1 (1.3)
Time of cessation of daily smoking	
I still smoke daily	76 (96.1)
2 days–6 days ago	1 (1.3)
1 month–less than 1 year ago	1 (1.3)
1–5 years ago	1 (1.3)
B. Biological Indicator of Smoking Exposure	
Average Exhaled CO	21.9 (8.3)
Exhaled CO	
7–10 Danger Zone	4 (5.0)
11–15 Smoker	15 (19.0)
16–25 Frequent smoker	38 (48.1)
26–35 Addicted smoker	15 (19.0)
36–50 Heavily Addicted Smoker	7 (8.9)
C: Perception and Experience of Smoking Cessation	
Number of attempts made to quit smoking	5.1 (12.4)
Number of serious quitting attempts in the past 2 years	1.7 (5.6)
Approaches to serious quitting attempts	
I never had a serious attempt to quit	29 (36.7)
I called a quit line	6 (7.6)
I quit cold turkey without any help or preparation	30 (38.0)
I quit cold turkey but I had it well planned and I set a quit date	12 (15.2)
I used a medication for smoking cessation (Nicotine gum, patch, pills)	2 (2.5)
Characterization of the last quitting attempt	
Very difficult	27 (34.1)
Difficult	21 (26.6)
Easy	15 (19.0)
Never tried	16 (20.3)
Ease of avoiding smoking in prohibited places	
Yes	28 (35.4)
No	51 (64.6)
Confidence in quitting attempt in 1 year	
1–5 (1 = not at all)	13 (16.5)
6–10 (10 = very much)	66 (83.5)
The main reason for smoking cessation/reduction	
The cost of tobacco products I use	7 (8.9)
To improve my sense of taste or smell	5 (6.3)
The messiness or dirtiness of the habit	6 (7.6)
The effect of smoking on my health	58 (73.4)
Having my doctor tell me to stop or cut down	1 (1.3)
Other	2 (2.5)
D: Secondhand Smoking	
Exposure to indoor tobacco smoke at home (other than the tobacco used by the participant)	
Yes	19 (24.1)
No	60 (75.9)
Measures applied at the current place of residence	
Smoking is strictly forbidden at home, I/smokers can’t smoke even in the balcony or porch	50 (63.3)
Smoking is forbidden inside the home, but I/smokers can smoke on the balcony or porch	18 (22.8)
Smoking is allowed inside the home, I/smokers can smoke on the balcony or porch	11 (13.9)
Presence of any other tobacco smokers in the household	
Yes	11 (13.9)
No	68 (86.1)

**Table 3:** Characteristics of participants lost to follow-up in comparison to the completed cases (N = 79).

Variables	Completed Cases Mean (SD) or *n* (%) N = 65	Loss to Follow Up Mean (SD) or *n* (%) N = 14	Significance (*p*-Value)
Age (in years)	43.0 (10.4)	44.1 (11.9)	0.74
Marital Status			
Married	52 (80)	12 (85.8)	
Divorced	3 (4.6)	1 (7.1)	
Never married	10 (15.4)	1 (7.1)	
Schooling (in years)	14.6 (3.2)	15.1 (6.5)	0.81
Employment			
Working for pay	53 (81.6)	9 (64.3)	
Unemployed and looking for work	8 (12.4)	4 (28.6)	
Temporarily laid off or on leave	1 (1.5 )	0 (0 )	
Disabled/Unable to work	1 (1.5)	0 (0)	
Retired	1 (1.5)	0 (0)	
Student	1 (1.5)	1 (7.1)	
Age when moved to the US	30.6 (13.8)	22.9 (11.7)	0.79
Number of daily smoked cigarettes	20.3 (9.2)	17.3 (8.7)	0.29
Number of cigarettes smoked in the past week	162.5 (85.9)	125.2 (68.6)	0.15
Number of attempts made to quit smoking	5.8 (13.6)	1.5 (1.2)	0.05
Number of quitting attempts in the past 2 years	1.95 (6.2)	0.7 (0.6)	0.2
Serious quitting attempts			
I never had a serious attempt to quit	26 (40.0)	3 (21.4)	
I called a quit line	3 (4.6)	3 (21.4)	
I quit cold turkey without any help or preparation	27 (41.5)	3 (21.4)	
I quit cold turkey but I had it well planned and I set a quit date	8 (12.4)	4 (28.7)	
Medication used for smoking cessation (Nicotine gum, patch, pills)	1 (1.5)	1 (7.1)	
Description of the last quitting attempt			0.87
Very difficult	23 (35.4)	4 (28.6)	
Difficult	16 (24.6)	5 (35.7)	
Easy	13 (20.0)	2 (14.3)	
Never tried	13 (20.0)	3 (21.4)	
Exhaled CO	21.9 (8.0)	21.9 (9.6)	0.85
Categories of Exhaled CO			
7–10 Danger Zone	3 (4.6)	1 (7.1)	
11–15 Smoker	12 (18.4)	3 (21.4)	
16–25 Frequent smoker	33 (50.8)	5 (35.7)	
26–35 Addicted smoker	12 (18.5)	3 (21.4)	
36–50 Heavily Addicted Smoker	5 (7.7)	2 (14.3)	
Total score of Fagerstrom Nicotine Dependence	12 (2.9)	11.7 (2.7)	0.76
Total score of Smoking Self-Efficacy Scale	11.1 (6.2)	16.4 (13.7)	0.21

**Table 4:** Loss to follow-up reflected in the Minnesota nicotine withdrawal scale (MNWS) (N = 79).

Variables	Completed Cases Mean (SD), N = 65	Loss to Follow-Up Mean (SD) N (Week 2) = 6 N (Week 4) = 2
**During Intervention—Week 2**		
Total Score MNWS	16.5 (4.8)	21.5 (7.8)
HR	82.3 (3.9)	81.5 (2.7)
Body Weight	80.9 (9.1)	74.2 (2.3)
**During Intervention—Week 4**		
Total Score MNWS	16.2 (6.8)	26 (15.6)
HR	82.3 (3.9)	82.5 (3.5)
Body Weight	80.9 (9.0)	75.5 (0.7)

**Table 5:** Smoking status and cessation before and after the *Sehatack* program (N = 65).

Variables	Baseline Mean (SD) or *n* (%) N = 65	Post Mean (SD) or *n* (%) N = 65
**Number of daily smoked cigarettes**	20.3 (9.2)	4.3 (6.3)
**Number of weekly smoked cigarettes**	162.5 (85.9)	30.6 (43.6)
**Desire to quit smoking**		
1–5 (1 = not at all)	12 (18.4)	0 (0)
6–10 (10 = very much)	53 (81.6)	65 (100)
**Confidence in quitting attempt in 1 year**		
1–5 (1 = not at all)	10 (15.3)	1 (1.5)
6–10 (10 = very much)	55 (84.7)	64 (98.5)
**Time of cessation of daily smoking**		
I still smoke daily		10 (15.4)
I smoke occasionally now but have not stopped yet		26 (40.0)
Today or yesterday		1 (1.5)
2 days–6 days ago		28 (43.1)
**Changed attitude towards smoking cessation**		
Yes, I am more interested in quitting		65 (100)
**Plan of smoking cessation for the following month**		
I will be quitting		22 (33.8)
I will reduce from current level		5 (7.7)
I will keep current level		9 (13.8)
I will maintain quitting (I do not smoke now)		29 (44.6)

**Table 6:** Smoking reduction (N = 79).

Intervention Status	I Still Smoke Daily N (%)	I Smoke Occasionally Now But Have Not Stopped Yet N (%)	Today or Yesterday N (%)	2 Days–6 Days Ago N (%)	Wilcoxon Signed Rank	*p*-Value
**Pre-intervention**	79 (49.37)	0 (0)	1 (0.63)	0 (0)	−8.8516	<0.0001
**Post-intervention**	23 (14.56)	26 (16.46)	2 (1.27)	28 (17.72)		

**Table 7:** Comparison for median for number of cigarettes per day and per weeks before and after *Sehatack* program (N = 79).

	Baseline Median (Q1–Q3) (N = 79)	Post-Intervention Median (Q1–Q3) (N = 79)	Wilcoxon Sign Ranked Test	*p*-Value
**On average, how many cigarettes do you smoke per day?**	20 (12–22)	3 (0–13)	−5.8391	<0.0001
**How many cigarettes did you smoke in the last 7 days?**	140 (80–210)	21 (1–70)	−8.1274	<0.0001

## Supplementary Materials

The following are available online at www.mdpi.com/1660-4601/14/4/411/s1, NRT Screening Tool and Dosing Guide.
