# Supplementary material for: Effectiveness of a Culturally-Tailored Smoking Cessation Intervention for Arab-American Men
Source: Int J Environ Res Public Health. 2017 Apr 13;14(4):411. doi: 10.3390/ijerph14040411 (PMC5409612; doi:10.3390/ijerph14040411)
Supplement: Supplementary file 1 [file ijerph-14-00411-s001.pdf]

Effectiveness of a Culturally-Tailored Smoking Cessation Intervention for Arab-American Men

Linda G. Haddad, Ahmad M. Al-Bashaireh, Anastasiya V. Ferrell and Roula Ghadban

Supplementary files: NRT Screening Tool and Dosing Guide

Participant #: ____________________

Date: ____________________

Cigarettes per day (CPD): ____________________

Quit date: ____________________

RA: ask the participant the following questions.

1. Have you been hospitalized for a heart-related condition in the past 2 weeks?

○ Yes ○ No

2. Have you had a heart attack (a myocardial infarction) in the past 2 weeks?

○ Yes ○ No

3. Are you pregnant or breastfeeding, or do you plan to become pregnant in the next year?

○ Yes ○ No

RA: Answer must be "No" to all 3 questions to dispense NRT. If participant answers "Yes" to any question, inform participants that they should consult with their doctor about use of NRT, and advise to try another method.

4. Have you ever tried any nicotine replacement therapy products, such as the nicotine patch, nicotine gum, nicotine inhaler, or nicotine lozenge?

○ Yes ○ No → offer monotherapy

If yes: Which have you tried? ________________

Was it helpful in reducing cravings? ○ Yes → offer monotherapy

---

NRT dispensed? ○ Yes ○ No

If yes—see dosing guide and note:

Nicotine patch dosage/weeks/boxes: ________________________

DOSING GUIDE FOR NRT

- All interested participants will be offered 6-10 total weeks of NRT (depending on CPD)
- All participants will be screened for contraindications
- All participants will be given specific directions at the first visit and 4-week visit, based on their CPD
- Choice of first line therapy will be based on prior experience and participant preference
- Subsequent quit attempts may involve combination therapies to address experiences during the past attempt

If participant has never used any NRT aids to help with quitting, start with one med by itself, typically the patch. If participant has used patch before and it was helpful in reducing cravings have him/her use patch again (even if previous quit attempt was unsuccessful **DOSING FOR PATCH ONLY:**

If greater than or equal to 10 CPD:

Dose	Time
21 mg	4 weeks
14 mg	2 weeks
7 mg	2 weeks

If less than 10 CPD:

Dose	Time
14 mg	4 weeks
7 mg	2 weeks

DOSING AT 4-week visit (for those who have already chosen NRT at Visit #1):

1) Continue current schedule (tapering dose as directed above) over the next 4-6 weeks
2) Continue with patch schedule Dose will be based on current CPD using worksheet

Modified patch dosing schedule for 6 weeks only, if greater than or equal to 10 CPD:

Dose	Time
21 mg	2 weeks
14 mg	2 weeks

7 mg	2 weeks

If less than 10 CPD, dosing schedule for patch only for patch can be the same as the original schedule, as it is only a 6-week course.
